# Small and Medium Amplitude Oscillatory Shear Rheology of Model Branched Polystyrene (PS) Melts

**DOI:** 10.3390/polym12020365

**Published:** 2020-02-07

**Authors:** Hyeong Yong Song, Lorenz Faust, Jinha Son, Mingeun Kim, Seung Joon Park, Suk-kyun Ahn, Manfred Wilhelm, Kyu Hyun

**Affiliations:** 1 School of Chemical and Biomolecular Engineering, Pusan National University, Busan 46241, Korea; diabloone1@pusan.ac.kr (H.Y.S.); 1o6b1o6b@gmail.com (M.K.); 2 Institute of Chemical Technology and Polymer Chemistry, Karlsruhe Institute of Technology (KIT), Engesserstraβe 18, 76131 Karlsruhe, Germany; lorenz.faust@kit.edu (L.F.); manfred.wilhelm@kit.edu (M.W.); 3 Department of Polymer Science and Engineering, Pusan National University, Busan 46241, Korea; sjinha2426@gmail.com (J.S.); skahn@pusan.ac.kr (S.-k.A.); 4 Department of Chemical Engineering and Biotechnology, Korea Polytechnic University, Siheung-Si, Gyeonggi-Do 15073, Korea; sjpark@kpu.ac.kr

**Keywords:** comb polymer, Bottlebrush polymer, medium amplitude oscillatory shear (MAOS), FT rheology, nonlinear parameter

## Abstract

Linear and nonlinear rheological properties of model comb polystyrenes (PS) with loosely to densely grafted architectures were measured under small and medium amplitude oscillatory shear (SAOS and MAOS) flow. This comb PS set had the same length of backbone and branches but varied in the number of branches from 3 to 120 branches. Linear viscoelastic properties of the comb PS were compared with the hierarchical model predictions. The model underpredicted zero-shear viscosity and backbone plateau modulus of densely branched comb with 60 or 120 branches because the model does not include the effect of side chain crowding. First- and third-harmonic nonlinearities reflected the hierarchy in the relaxation motion of comb structures. Notably, the low-frequency plateau values of first-harmonic MAOS moduli scaled with $M_{w}^{-2}$ (total molecular weight), reflecting dynamic tube dilution (DTD) by relaxed branches. Relative intrinsic nonlinearity *Q*_0_ exhibited the difference between comb and bottlebrush via no low-frequency *Q*_0_ peak of bottlebrush corresponding to backbone relaxation, which is probably related to the stretched backbone conformation in bottlebrush.

## 1. Introduction

Understanding the rheological properties of branched polymers is essential for all forms of production and processing in the polymer industry because the processability of polymers is highly affected by the degree of long-chain branches (LCB) [1]. A branched polymer with LCB exhibits remarkable linear and nonlinear rheological properties, such as hierarchical relaxation, weak shear thinning with faster onset and strain hardening at low rates in elongation [2,3,4,5,6]. However, full characterization of the effect of LCB on rheological properties is still challenging because it requires detailed molecular information of molecular weight and polydispersity of branches, the density of branch points and the distribution of branches along the backbone or other branches [7]. Instead, many researches have used well-defined model branched polymers of star [8], pom-pom [9], comb [10] and dendritic structures [11]. Architectures of these polymers are a substructure of industrial polymers with LCB.

Comb polymer is the simplest class of model branched polymer with multiple branch points. Generally, it consists of a linear backbone and multiple side branches [12]. Comb architecture is an important chain structure because metallocene-catalyzed synthesis can lead to sparsely branched comb polymers [13]. The fundamental concept of the comb polymer is the hierarchy in relaxation motions [6]. In other words, the backbone chain can relax after the branches relax completely. This hierarchical relaxation process affects both linear and nonlinear rheology of comb polymers. Kapnistos, et al. [14] reported that linear rheology of comb polystyrenes (PS) with many entangled branches exhibited two rubbery plateaus, one relating to the branch relaxation and the other relating to the backbone relaxation. Nonlinear start-up shear measurements of these comb PS exhibited double stress overshoot behavior at shear rates exceeding the inverse of branch relaxation time [15]. In particular, the first overshoot was associated with the orientation of branches, while the second overshoot was associated with the stretch of backbone. Medium amplitude oscillatory shear (MAOS) measurements, one of the nonlinear shear tests, also reflected the hierarchical relaxation of the comb PS when relative intrinsic nonlinearity *Q*_0_ was plotted as a function of excitation frequency [16]. Two distinct *Q*_0_ peaks corresponded to branch relaxation at a higher frequency and backbone relaxation at a lower frequency.

The conformation of comb structure can be controlled by tuning the grafting density and the degree of polymerization of the side branches. Sheiko and coworkers [17,18,19] identified four conformational regimes in terms of these two parameters using scaling analysis: loosely grafted comb (LC), densely grafted comb (DC), loosely grafted bottlebrush (LB) and densely grafted bottlebrush (DB). Bottlebrush polymers have comb-like structures with a very high density of branches, usually incorporating up to one grafted side chains on every backbone repeat unit [20,21]. A significant difference between comb and bottlebrush polymers is the backbone conformation. The backbone of the bottlebrush is stretched by side chain crowding, whereas comb polymers have unperturbed Gaussian backbone and branches [17,18,19]. Recently, Abbasi, Faust, Riazi and Wilhelm [21] synthesized a series of well-defined comb and bottlebrush PS where the same backbone length was used but the number of entangled branches was systematically changed. They also investigated the effect of the number of branches on the linear viscoelastic (zero-shear viscosity) and nonlinear viscoelastic properties (extensional viscosity and strain hardening factor) of the model comb PS set. The results showed that a relative minimum in the zero-shear viscosity and a maximum in strain hardening factor were observed near a transition point from DC to LB, which was associated with the stretched backbone conformation grafted with crowding side chains [21,22]. Up to now, most rheological studies on bottlebrush polymers have focused on linear shear flow [20,23,24] and extensional flow [25,26]. There has been no report on nonlinear shear rheology.

In the current study, we investigated the MAOS behavior of the comb and bottlebrush PS used in Abbasi, Faust, Riazi and Wilhelm [21]. Recently, MAOS rheology has been used to characterize different polymer architectures, such as linear [27,28], 3-arm star [29] and comb [16,30]. When the relative intrinsic nonlinearity *Q*_0_, one of MAOS material parameters, was plotted as a function of excitation frequency, it showed a different number of local peaks depending on the number of terminal relaxation processes in polymer architectures. In particular, Hyun and Wilhelm [16] plotted *Q*_0_ master curves of comb PS to investigate the effect of branch length on *Q*_0_ at a fixed number of branches (~30). Reversely, we present the effect of the number of branches on *Q*_0_ at a fixed branch length. Furthermore, we present MAOS results of a bottlebrush polymer for the first time.

The rest of the paper is organized as follows: In Section 2, we outline the theoretical background and provide the definitions of MAOS material functions. Molecular characteristics of the model comb PS used are summarized in Section 3. Section 4 consists of four contents: (1) summary of linear viscoelastic data obtained from small amplitude oscillatory shear (SAOS) tests, (2) prediction results of the hierarchical model, (3) characterization of MAOS behavior of the comb PS set and (4) comparison of SAOS and MAOS material parameters in terms of characteristic relaxation times.

## 2. Definition of MAOS Material Functions

A sinusoidal strain excitation of amplitude *γ*_0_ and frequency *ω* [*γ*(*t*) = *γ*_0_ sin(*ωt*)] generates an oscillatory shear stress response *σ*(*t*). The obtained stress response can be decomposed into higher harmonic contributions via Fourier transformation as follows [31,32]:

(1) $$\sigma \left(t\right)=\gamma_{0}\sum_{n=1,\mathrm{odd}}^{\infty }\left\{{G^{\prime }}_{n}\left(\omega ,\gamma_{0}\right)\sin\left(n\omega t\right)+{G^{\prime \prime }}_{n}\left(\omega ,\gamma_{0}\right)\cos\left(n\omega t\right)\right\},$$

where $G_{n}^{\prime }$(*ω*, *γ*_0_) and $G_{n}^{\prime \prime }$(*ω*, *γ*_0_) are the Fourier moduli. The Fourier moduli can be represented by power series expansions in higher powers of *γ*_0_ [33,34]. The first- and third-harmonic Fourier moduli are expanded as follows:

(2) $$\begin{array}{l} {G^{\prime }}_{1}\left(\omega ,\gamma_{0}\right)=G^{\prime }\left(\omega \right)+{G^{\prime }}_{31}\left(\omega \right)\gamma_{0}^{2}+O\left(\gamma_{0}^{4}\right)\\ {G^{\prime \prime }}_{1}\left(\omega ,\gamma_{0}\right)=G^{\prime \prime }\left(\omega \right)+{G^{\prime \prime }}_{31}\left(\omega \right)\gamma_{0}^{2}+O\left(\gamma_{0}^{4}\right)\\ {G^{\prime }}_{3}\left(\omega ,\gamma_{0}\right)={G^{\prime }}_{33}\left(\omega \right)\gamma_{0}^{2}+O\left(\gamma_{0}^{4}\right)\\ {G^{\prime \prime }}_{3}\left(\omega ,\gamma_{0}\right)={G^{\prime \prime }}_{33}\left(\omega \right)\gamma_{0}^{2}+O\left(\gamma_{0}^{4}\right) \end{array}$$

Therefore, Equation (1) can be re-written up to the third harmonic as

(3) $$\begin{array}{ll} \sigma \left(t\right)= & \gamma_{0}\left\{G^{\prime }\left(\omega \right)\sin\left(\omega t\right)+G^{\prime \prime }\left(\omega \right)\cos\left(\omega t\right)\right\}\\ & +\gamma_{0}^{3}\left\{\begin{array}{l} {G^{\prime }}_{31}\left(\omega \right)\sin\left(\omega t\right)+{G^{\prime \prime }}_{31}\left(\omega \right)\cos\left(\omega t\right)\\ +{G^{\prime }}_{33}\left(\omega \right)\sin\left(3\omega t\right)+{G^{\prime \prime }}_{33}\left(\omega \right)\cos\left(3\omega t\right) \end{array}\right\}+O\left(\gamma_{0}^{5}\right) \end{array}$$

In Equation (3), the linear amplitude scaling term represents linear viscoelastic or SAOS response, characterized by two SAOS moduli [*G*′(*ω*) and *G*″(*ω*)]. The cubic amplitude scaling term represents medium amplitude oscillatory shear (MAOS) response, characterized by four MAOS moduli [$G_{31}^{\prime }$(*ω*), $G_{31}^{\prime \prime }$(*ω*), $G_{33}^{\prime }$(*ω*) and $G_{33}^{\prime \prime }$(*ω*)]. The first-harmonic MAOS moduli [$G_{31}^{\prime }$(*ω*) and $G_{31}^{\prime \prime }$(*ω*)] affect asymptotic deviations in average (i.e., intercycle) elasticity or viscosity by causing rotations of elastic or viscous Lissajous curves [35,36]. The third-harmonic moduli, which have been termed intracycle nonlinearities, generate local deviations from linear viscoelastic stress within an oscillation cycle [35,37]. Under MAOS flow, harmonic contributions higher than fifth are ignored and thus MAOS response can be reconstructed by two SAOS moduli and four MAOS moduli.

Another MAOS material parameter can be obtained from the Fourier-transform (FT) rheology framework. The relative intensity of the third harmonic *I*_3/1_(*ω*, *γ*_0_) is defined using the first- and third-harmonic Fourier moduli [16].

(4) $$\begin{array}{ll} I_{3/1}\left(\omega ,\gamma_{0}\right) & \equiv \frac{\left|G_{3}^{*}\right|}{\left|G_{1}^{*}\right|}=\frac{\sqrt{{G^{\prime }}_{3}^{2}+{G^{\prime \prime }}_{3}^{2}}}{\sqrt{{G^{\prime }}_{1}^{2}+{G^{\prime \prime }}_{1}^{2}}}\\ & =\frac{\sqrt{{\left\{{G^{\prime }}_{33}\gamma_{0}^{2}+O\left(\gamma_{0}^{4}\right)\right\}}^{2}+{\left\{{G^{\prime \prime }}_{33}\gamma_{0}^{2}+O\left(\gamma_{0}^{4}\right)\right\}}^{2}}}{\sqrt{{\left\{G^{\prime }+{G^{\prime }}_{31}\gamma_{0}^{2}+O\left(\gamma_{0}^{4}\right)\right\}}^{2}+{\left\{G^{\prime \prime }+{G^{\prime \prime }}_{31}\gamma_{0}^{2}+O\left(\gamma_{0}^{4}\right)\right\}}^{2}}}\\ & =\frac{\sqrt{{G^{\prime }}_{33}^{2}+{G^{\prime \prime }}_{33}^{2}+O\left(\gamma_{0}^{2}\right)}}{\sqrt{{G^{\prime }}^{2}+{G^{\prime \prime }}^{2}+O\left(\gamma_{0}^{2}\right)}}\cdot \gamma_{0}^{2} \end{array}$$

Equation (4) shows the quadratic amplitude scaling of *I*_3/1_ under the MAOS flow. Using this scaling relationship, an intrinsic nonlinearity *Q*_0_(*ω*) is defined as [16]

(5) $$Q_{0}\left(\omega \right)\equiv \underset{\gamma_{0}\to 0}{\lim}\frac{I_{3/1}\left(\omega ,\gamma_{0}\right)}{\gamma_{0}^{2}}=\frac{\sqrt{{G^{\prime }}_{33}^{2}+{G^{\prime \prime }}_{33}^{2}}}{\sqrt{{G^{\prime }}^{2}+{G^{\prime \prime }}^{2}}}=\frac{\left|G_{33}^{*}\right|}{\left|G^{*}\right|}.$$

By definition, *Q*_0_ is related to the third-harmonic MAOS moduli. Because *Q*_0_ is a combined measure of two third-harmonic MAOS moduli, *Q*_0_ can be decomposed into elastic and viscous parts as follows [37]:

(6) $${Q^{\prime }}_{0}\left(\omega \right)\equiv \frac{{G^{\prime }}_{33}}{\sqrt{{G^{\prime }}^{2}+{G^{\prime \prime }}^{2}}}=\frac{{G^{\prime }}_{33}}{\left|G^{*}\right|}$$

(7) $${Q^{\prime \prime }}_{0}\left(\omega \right)\equiv \frac{{G^{\prime \prime }}_{33}}{\sqrt{{G^{\prime }}^{2}+{G^{\prime \prime }}^{2}}}=\frac{{G^{\prime \prime }}_{33}}{\left|G^{*}\right|}.$$

A detailed discussion of MAOS material functions can be found in a recent publication of Song and Hyun [38].

## 3. Materials and Methods

### 3.1. Materials

Model PS combs used were synthesized by anionic polymerization [21]. Detailed molecular characteristics are summarized in Table 1. Molecular weights and polydispersity of backbone, branch and combs were determined using size exclusion chromatography equipped with multi angle laser light scattering (SEC-MALLS). Abbasi, Faust, Riazi and Wilhelm [21] quantified the number of branches per backbone chain (*N*_br_) by three different methods—SEC-MALLS, SEC and ^1^H nuclear magnetic resonance (NMR). Here, we used the values for *N*_br_ determined by SEC-MALLS because this method is more accurate for a comb with a high molecular weight [21]. The details for synthesis strategy and characterization techniques were described in Abbasi, Faust, Riazi and Wilhelm [21].

Topologies of comb and bottlebrush polymers were determined using scaling analysis [17,18,19]. The scaling analysis subdivided comb and bottlebrush structures into four conformational regimes: loosely grafted comb (LC), densely grafted comb (DC), loosely grafted bottlebrush (LB) and densely grafted bottlebrush (DB). Abbasi, Faust, Riazi and Wilhelm [21] determined topologies of the synthesized comb PS set using the scaling analysis, which are listed in Table 1.

### 3.2. Rheological Measurements

We used a strain-controlled rotational rheometer (ARES-G2, TA Instruments) for measurements of SAOS and MAOS material functions. SAOS and MAOS measurements were performed using a parallel plate (PP) geometry with a diameter of 13 mm under a nitrogen environment to prevent oxidative degradation of samples. Master curves of SAOS and MAOS material functions were obtained using the time-temperature superposition (TTS) principle at a reference temperature (*T*_ref_) of 180 °C. MAOS material functions measured using PP geometry need to be corrected by multiplying a vertical shift factor of 1.5 to compensate for the inhomogeneous flow field [39]. However, in the current study, uncorrected values were used without a vertical shift because we did not compare MAOS experimental data with a theoretical model.

## 4. Results and Discussion

### 4.1. SAOS Results

The linear viscoelastic behavior of model comb PS used has already been analyzed by Abbasi, Faust, Riazi and Wilhelm [21]. Here, we introduce key results briefly and then discuss the prediction results of the hierarchical model developed by Larson and coworkers [7,40,41].

**Figure 1:**
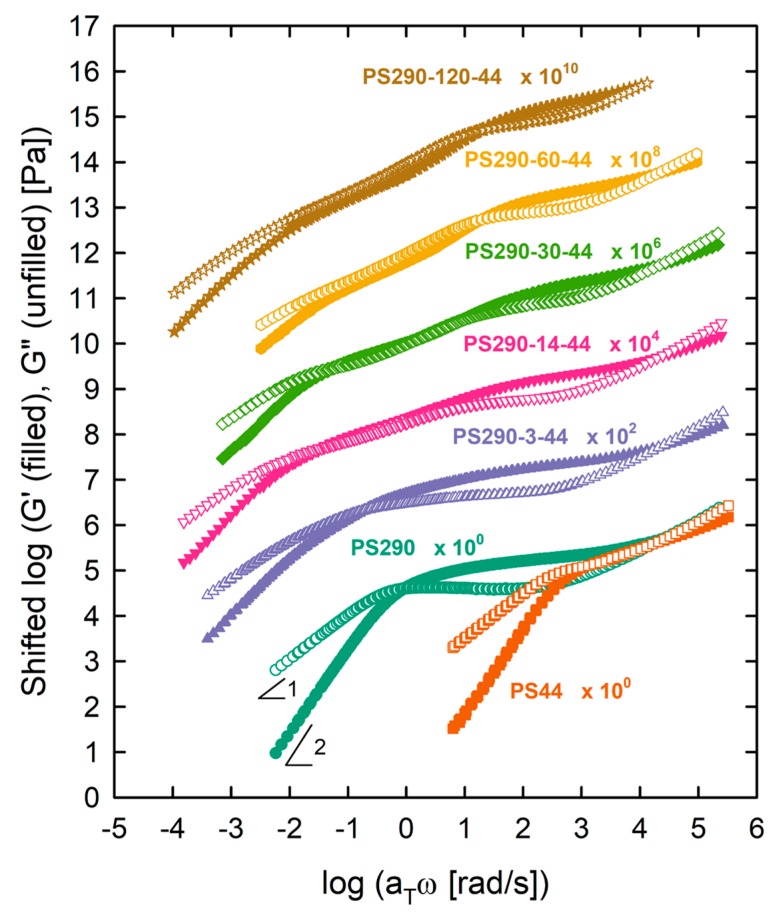
Linear master curves of model comb PS set at *T*_ref_ = 180 °C. Moduli were shifted vertically for clarity of presentation.

Figure 1 shows the linear master curves of *G*′(*ω*) and *G*″(*ω*) at *T*_ref_ = 180 °C. LC and DC PS with 3 ≤ *N*_br_ ≤ 30 displayed a crossover of *G*′ and *G*″ at low frequency, indicating the reptation dynamics of the backbone chain. In contrast, DC and LB with *N*_br_ ≥ 60 showed a power-law behavior of *G*′ and *G*″ (~ *ω*^0.6^), indicating the Rouse dynamics of the backbone chain. Basically, comb polymers relax hierarchically [6,14]. After the relaxation of branches by retraction toward the branch points, the relaxed branches act as effective solvents for the backbone and thus the backbone tube swells. This is called the dynamic tube dilution (DTD) effect. As a result, the backbone is free to relax in a widened tube. Relating to this, there are two rubbery plateaus. One at higher frequency corresponded to the branch relaxation but the other at lower frequency corresponded to the relaxation of the diluted backbone [14]. As the grafting density of branches increased, the diameter of the backbone tube became wider and as a result, the terminal relaxation of the backbone changed from reptation to Rouse-like behavior.

Abbasi, Faust, Riazi and Wilhelm [21] plotted the zero-shear viscosity *η*_0_ and the backbone plateau modulus *G*_N,bb_ of all comb PS as functions of the total molecular weight *M*_w_ (Figure 4 in this study). The DTD effect of the relaxed branches reduced the *G*_N,bb_. The diluted *G*_N,bb_ was proportional to $M_{w}^{-2}$, which was in agreement with *G*_N,bb_ ~ $\phi_{\mathrm{bb}}^{1+\alpha }$, where the dynamic dilution exponent *α* = 1 and the backbone volume fraction $\phi_{\mathrm{bb}}$ = *M*_w,bb_/*M*_w_. Interestingly, *η*_0_ exhibited different behavior in each conformational regime. LC PS behaved like star molecules where *η*_0_ increased exponentially as a function of *M*_w_ (*η*_0_ ~ exp (*M*_w_)), which is related to the strong frictional effect of the branches on the backbone [22]. A further increase of *N*_br_ resulted in a continuous decrease of *η*_0_, reflecting the dilution effect of the branches. The *η*_0_ of DC PS followed the scaling behavior of entangled or unentangled linear chains. The DC PS with 6 ≤ *N*_br_ ≤ 20 showed a power-law dependence of *η*_0_ on *M*_w_ with a scaling exponent of –3.4 (*η*_0_ ~ $M_{w}^{-3.4}$) corresponding to the entangled chain dynamics (reptation combined with contour length fluctuation (CLF) and constraint release (CR)). For DC PS with 20 ≤ *N*_br_ ≤ 142, *η*_0_ decreased with a power of −1 as a function of *M*_w_. This indicated that the backbone chain relaxed finally with a Rouse mechanism of unentangled chains. The change of *η*_0_ behavior was also related to the average entanglement number of backbone segments between the neighboring branch points (*Z*_s_), listed in Table 1 (*η*_0_ ~ $M_{w}^{-3.4}$ for DC PS with *Z*_s_ > 1 but *η*_0_ ~ $M_{w}^{-1}$ for DC PS with *Z*_s_ < 1). As chain conformation changed from DC to LB, *η*_0_ increased dramatically again, with the scaling exponent of about 5 (*η*_0_ ~ $M_{w}^{5}$). This strong dependence of *η*_0_ was related to the intramolecular interactions between neighboring entangled side branches due to the tight spacing between branch points.

### 4.2. Hierarchical Modeling

Based on these findings, we predicted the linear viscoelastic behavior of the model comb PS set using the hierarchical model [41]. Currently, the linear viscoelastic behavior of branched polymers can be reconstructed quantitatively using the three state-of-the-art tube-based models, that is, hierarchical model [7,40], branch-on-branch (BoB) model [42] and time-marching algorithm (TMA) [43]. Abbasi, Faust, Riazi and Wilhelm [21] commented that these models do not include an additional interaction originating from the tightly spaced branches (*Z*_s_ < 1). We used the hierarchical model to present how the model fails to predict the responses of comb PS with *Z*_s_ < 1. The parameters needed in the hierarchical model are the plateau modulus *G*_N_, the entanglement molecular weight *M*_e_, the equilibration time *τ*_e_ and the dynamic dilution exponent *α*. For PS, we used *G*_N_ = 2 × 10^5^ Pa, *M*_e_ = 14.4 kg/mol, *τ*_e_ = 3 × 10^−4^ s and *α* = 1 at *T*_ref_ = 180 °C.

**Figure 2:**
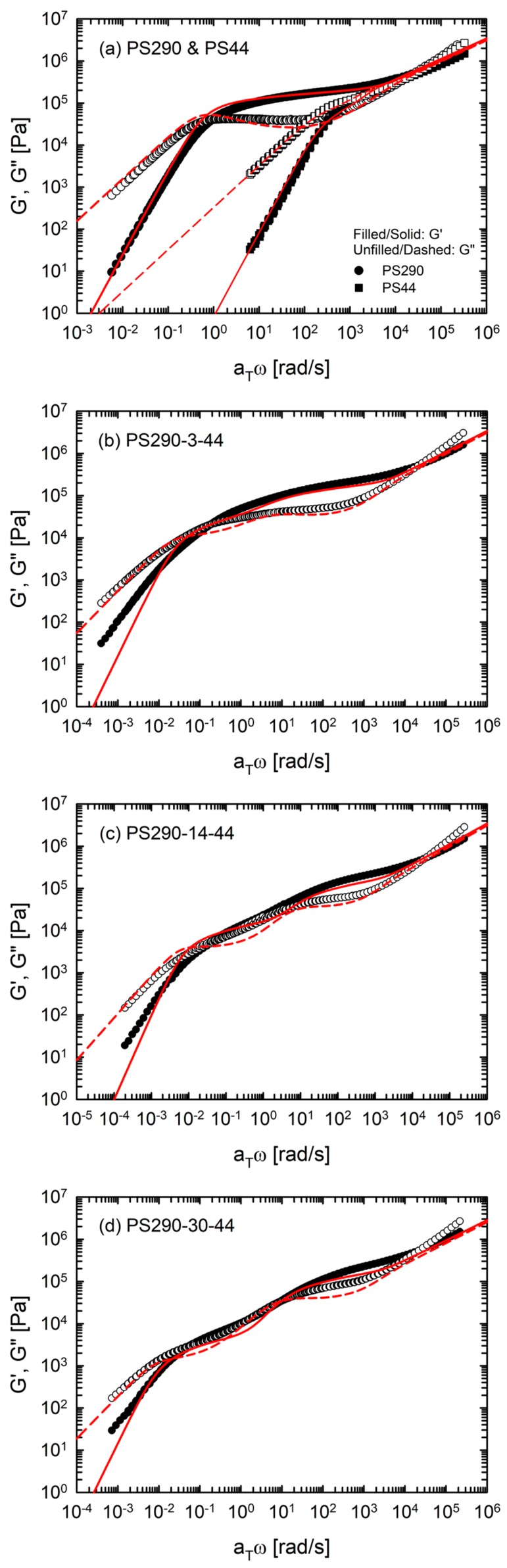

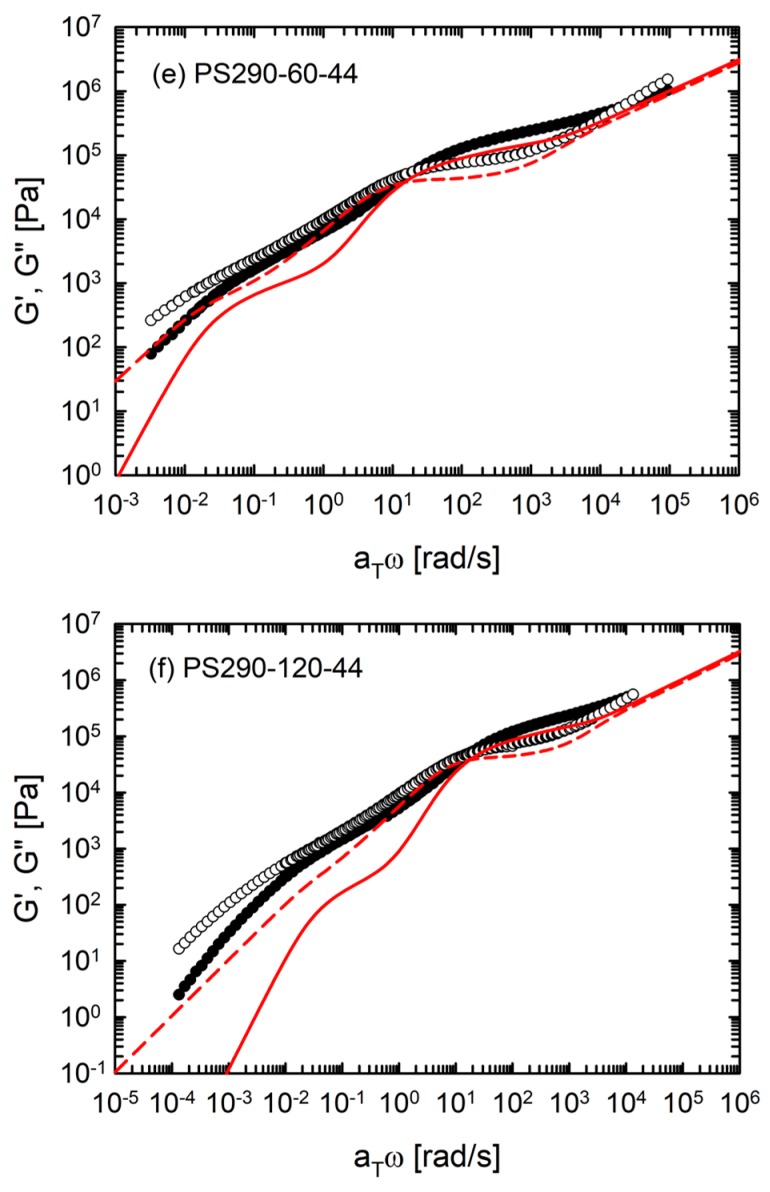
Comparison between experimental data and the hierarchical model predictions. (**a**) PS290, PS44 (**b**) PS290-3-44, (**c**) PS290-14-44, (**d**) PS290-30-44, (**e**) PS290-60-44 and (**f**) PS290-120-44. The symbols indicate experimental data of the model comb PS. The lines indicate the predictions of the hierarchical model.

In Figure 2, we compare the predictions of the hierarchical model with experimental data of the model comb PS. The hierarchical model predicted the *G*′ and *G*″ of two linear chains (PS290 and PS44) quantitatively. The model also predicted the linear viscoelastic behavior of the comb PS well, with 3 ≤ *N*_br_ ≤ 30. However, for PS290-3-44 and PS290-14-44, deviations between the predictions and experimental data were observed in two respects. The overall curve shapes of experimental data are smooth and broad, compared with sharp shapes in the predictions. In addition, the experimental *G*′ exhibited the terminal slope (=2) at a lower frequency than the model *G*′ whereas *G*″ of the experimental data and the model coincided with each other. We note that the model *G*′ of PS290-3-44 (LC) showed two rubbery plateaus clearly, which was in contrast with one broad plateau in the experimental *G*′. This broad plateau was associated with the statistical distribution of branch points [21]. The high amount of reaction sites for side chains and its statistical distribution generated during synthesis might have led to asymmetric comb structure, rather than symmetric comb structure. Furthermore, the relation between the standard deviation of molecular weight and the polydispersity index indicated that *N*_br_ of PS290-3-44 is 3 ± 3 [21]. Taken together, PS290-3-44 might be a mixture of asymmetric or symmetric combs with an average of 3 branches.

**Figure 3:**
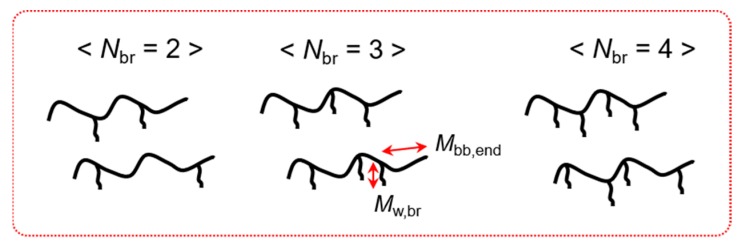
Possible structures of PS290-3-44. *M*_w,br_ is the branch molecular weight and *M*_bb,end_ is the molecular weight of the backbone end part.

Figure 3 shows several example structures of PS290-3-44 based on the above analysis. Kapnistos, Vlassopoulos, Roovers and Leal [14] demonstrated that the end parts of the linear backbone (*M*_bb,end_) behave like branches, dynamically diluting the backbone. If the branches are longer than the backbone end (*M*_w,br_ > *M*_bb,end_), the backbone end relaxes faster than the branches and the remaining backbone is immobile until the branches are fully retracted. On the other hand, if the branches are shorter than the backbone end (*M*_w,br_ < *M*_bb,end_), the branches relax first and the remaining unrelaxed part of the backbone end is added to the backbone. Therefore, the effective backbone length, the backbone length after the relaxation of the branches and the backbone ends, becomes longer in the latter case. If two outermost branches are located closer to the center of the backbone, *M*_w,br_ can be smaller than *M*_bb,end_. Thus, the uneven distribution of branch points results in different lengths of the effective backbone chain. Furthermore, the location of branch points can modify the terminal relaxation process of the effective backbone chain. Chen and Larson [44] demonstrated the effect of branch point position on the linear viscoelasticity of two asymmetric 3-arm star polymers (T-shaped and Y-shaped). After the arm relaxed, the length of the effective backbone was identical. However, Y-shaped polymer relaxed slower than T-shaped polymer because the branching point was located at the end of the effective backbone, where the frictional drag contributed from the branch retarded the terminal relaxation process of Y-shaped star. Consequently, distribution in the number and location of the branches results in different lengths of the effective backbone as well as different total frictional drag contributed from the branches, which made the relaxation spectrum of PS290-3-44 broad. On increasing *N*_br_, the effect of branch distribution becomes weaker and weaker because the branches are evenly attached to the backbone due to the steric effect. Under the assumption that branch points are evenly distributed along the backbone, the branch always becomes longer than the end part of the backbone when *N*_br_ ≥ 7.

The hierarchical model gave the best prediction for PS290-30-44. The terminal-regime predictions for PS290-30-44 coincided with the experimental *G*′ and *G*″. On the other hand, the model failed to predict the terminal behavior of PS290-60-44 and PS290-120-44. The predictions of PS290-60-44 and PS290-120-44 showed lower plateau modulus of backbone and earlier terminal behavior than the experimental values.

**Figure 4:**
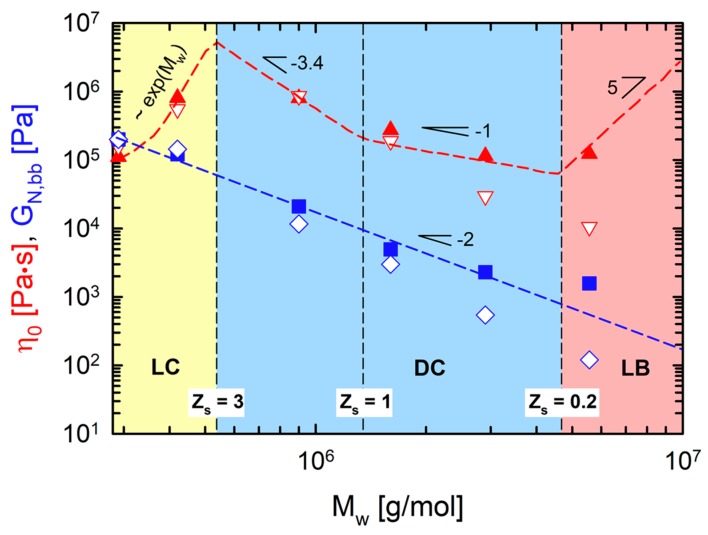
Zero-shear viscosity *η*_0_ and diluted backbone plateau modulus *G*_N,bb_ as functions of the total molecular weight *M*_w_. Filled symbols are the experimental data while unfilled symbols are the model predictions. Dashed lines are drawn to guide the eye.

For quantitative comparison, *η*_0_ and *G*_N,bb_ of the experimental data and the model predictions were plotted as functions of *M*_w_ (Figure 4). The model values well matched the experimental values until *N*_br_ = 30 (PS290-30-44). However, a further increase of *N*_br_ enhanced the DTD effect in the model, which resulted in lower *η*_0_ and *G*_N,bb_ predictions for PS290-60-44 and PS290-120-44. The model does not include the effect of side chain crowding when *Z*_s_ < 1. Thus, this deviation between the experimental data and the model originates from the densely branched topologies, where the backbone segments between the branch points are smaller than one entanglement segment [21].

### 4.3. MAOS Results

**Figure 5:**
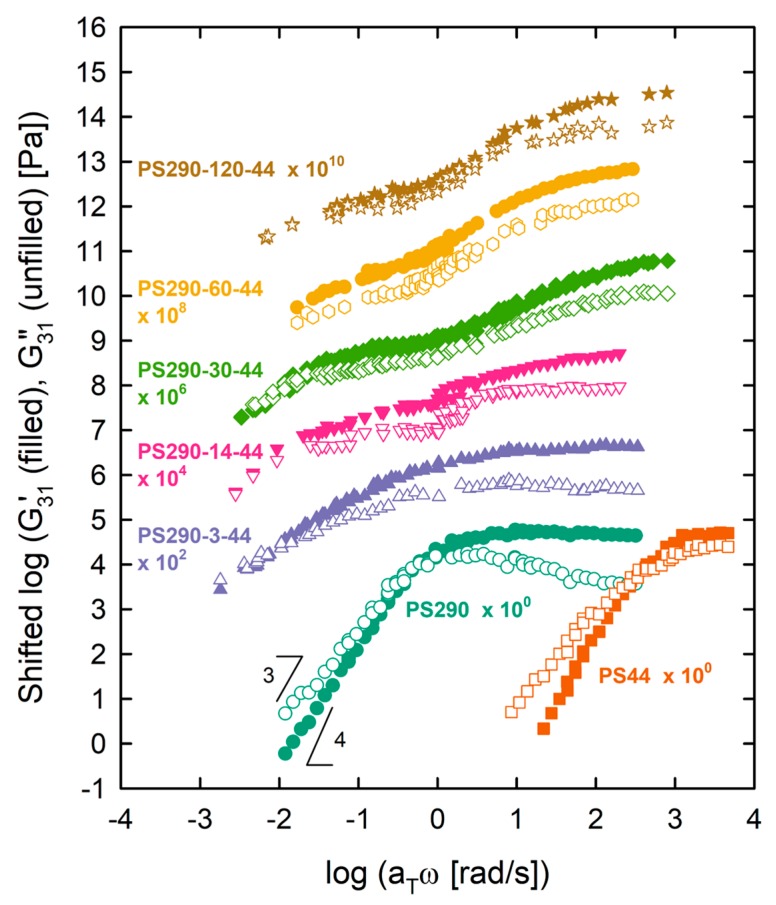
First-harmonic medium amplitude oscillatory shear (MAOS) moduli ($G_{31}^{\prime }$ and $G_{31}^{\prime \prime }$ of model comb PS set at *T*_ref_ = 180 °C. All values are negative. Moduli were shifted vertically for clarity of presentation.

Only four nonlinear moduli are required to reconstruct nonlinear response in the MAOS regime [35,38]. In this section, we present nonlinear master curves of the first- and third-harmonic MAOS moduli of model comb PS set. Figure 5 shows master curves of first-harmonic MAOS moduli ($G_{31}^{\prime }$ and $G_{31}^{\prime \prime }$) at *T*_ref_ = 180 °C. The first-harmonic MAOS moduli of all samples were negative, indicating intercycle strain softening and shear thinning of LAOS type Ⅰ [36,45]. The first-harmonic elastic MAOS moduli ($G_{31}^{\prime }$) of PS290 and PS44 exhibited the behavior of monodisperse linear chains reported by Song and Hyun [36]. In the rubbery plateau regime of SAOS data, $G_{31}^{\prime }$ also had a plateau value of about 5 × 10^4^ Pa. Compared with two linear PS, PS290-3-44 showed a broad curve shape due to a wide relaxation time distribution originating from polydispersity in the number and location of branch points. All DC and LB PS exhibited similar curve shapes of $G_{31}^{\prime }$ and $G_{31}^{\prime \prime }$, which contrasted with gradual shape developments in SAOS master curves on increasing *N*_br_. In addition, DC and LB showed two plateaus in $G_{31}^{\prime }$ and $G_{31}^{\prime \prime }$, like two rubbery plateaus in *G*′. The onset of further increase after the low-frequency plateau was observed near 1 rad/s, where branch retraction is finished [21]. This indicates that the first-harmonic MAOS moduli were affected by hierarchical relaxation processes. The low-frequency plateau corresponded to the backbone response and the high-frequency plateau corresponded to the branch response.

**Figure 6:**
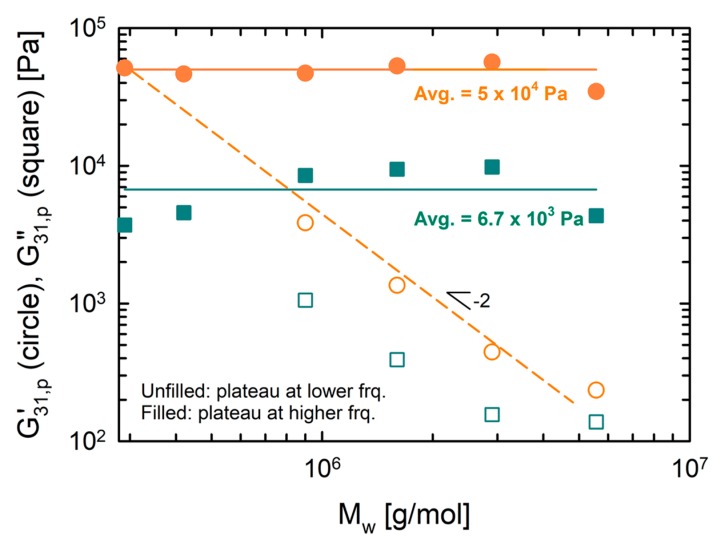
Plateau values of first-harmonic MAOS moduli at lower or higher frequencies ($G_{31,p}^{\prime }$ and $G_{31,p}^{\prime \prime }$ as a function of total molecular weight *M*_w_. Filled symbols indicate the values at a higher frequency. Unfilled symbols indicate the values at a lower frequency. The dashed line is drawn to guide the eye.

A further manifestation of the dilution effect on first-harmonic MAOS moduli is evidenced in Figure 6, which depicts plateau values of $G_{31}^{\prime }$ and $G_{31}^{\prime \prime }$ as a function of *M*_w_. The high-frequency plateau of $G_{31}^{\prime }$ (≡$G_{31,p}^{\prime }$) was constant as 5 × 10^4^ Pa while the low-frequency $G_{31,p}^{\prime }$ scaled with $M_{w}^{-2}$. The high-frequency plateau of $G_{31}^{\prime \prime }$ (≡$G_{31,p}^{\prime \prime }$) was averagely 6.7 × 10^3^ Pa. The low-frequency $G_{31,p}^{\prime \prime }$ of three DC PS also exhibited the same scaling behavior (~$M_{w}^{-2}$). These changes of $G_{31}^{\prime }$ and $G_{31}^{\prime \prime }$ are in accordance with the trend in SAOS plateau modulus (see Figure 4).

To check that the hierarchical relaxation affects a linear-to-nonlinear transition, we plotted the Pipkin diagram using the first-harmonic MAOS moduli. The critical strain amplitudes for the transition were calculated using the following equations [35,38]:

(8) $$\gamma_{0,e}^{*}\left(\omega \right)={\left|0.1\frac{{G^{\prime }}_{11}\left(\omega \right)}{{G^{\prime }}_{31}\left(\omega \right)}\right|}^{0.5}$$

(9) $$\gamma_{0,v}^{*}\left(\omega \right)={\left|0.1\frac{{G^{\prime \prime }}_{11}\left(\omega \right)}{{G^{\prime \prime }}_{31}\left(\omega \right)}\right|}^{0.5}.$$

**Figure 7:**
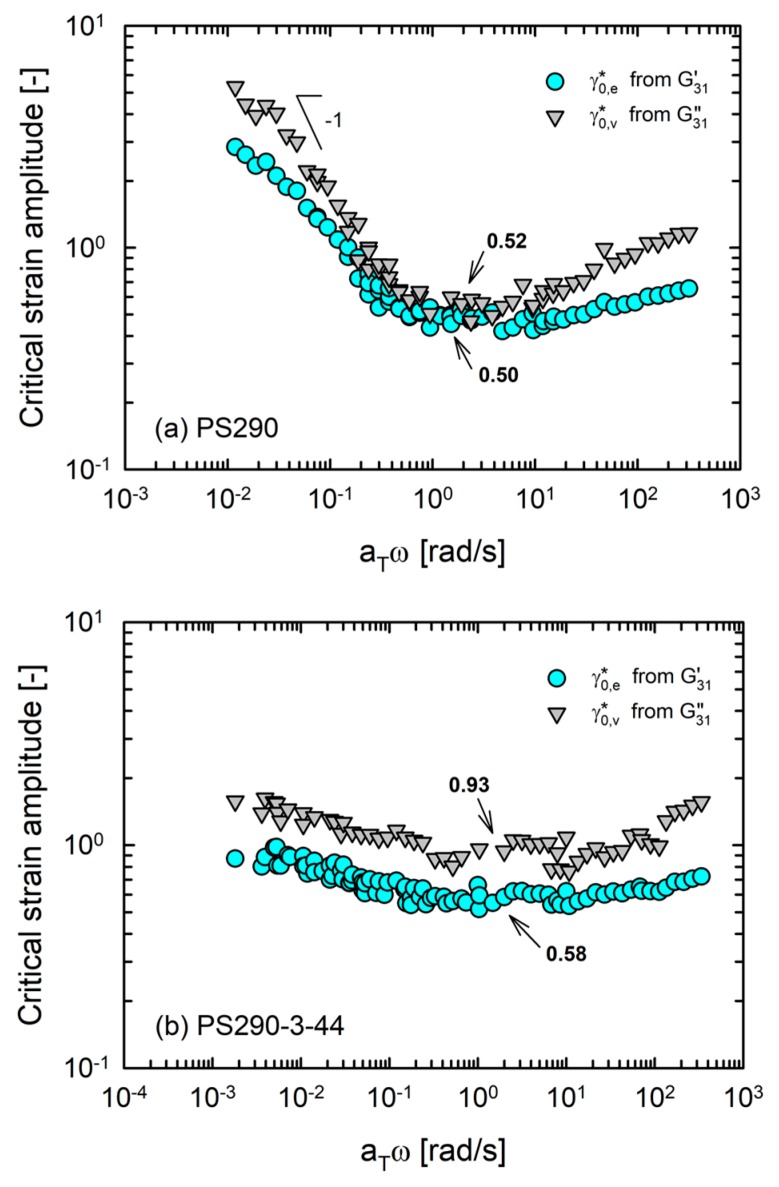

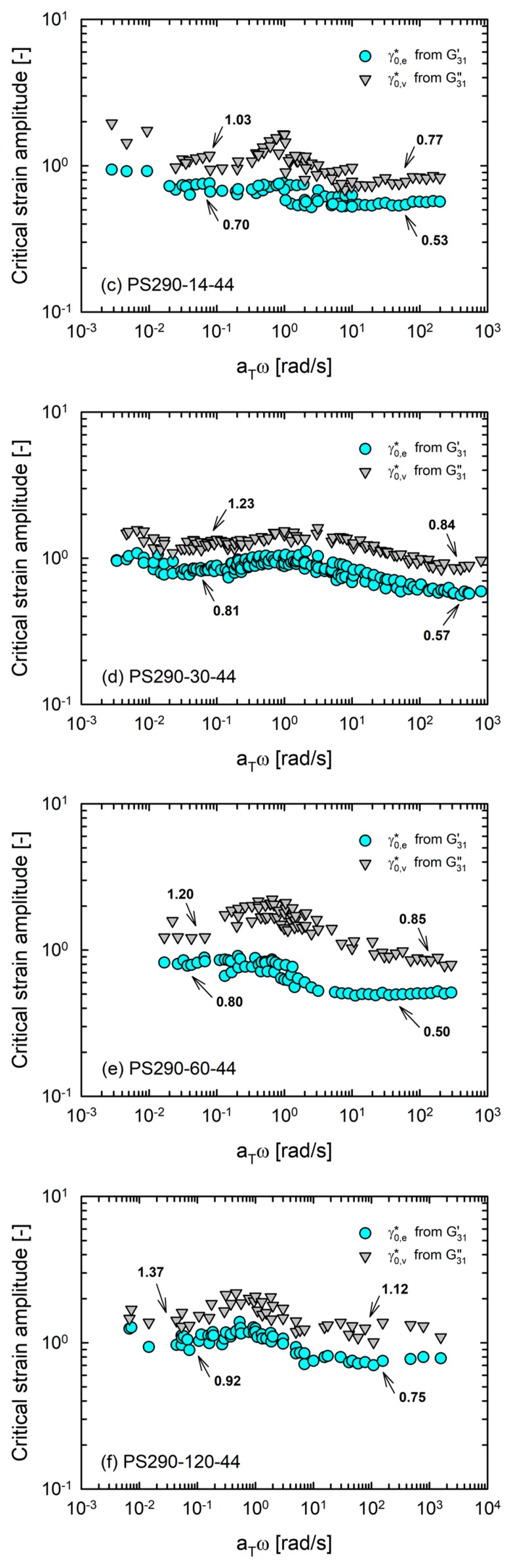
Critical strain amplitudes of model comb PS set as functions of frequency (Pipkin diagram) based on first-harmonic MAOS moduli. (**a**) PS290, (**b**) PS290-3-44, (**c**) PS290-14-44, (**d**) PS290-30-44, (**e**) PS290-60-44 and (**f**) PS290-120-44. The values represent the local minima in the plots.

We set a criterion for the MAOS limit as a 10% deviation from the linear viscoelasticity [35]. Figure 7 shows the Pipkin diagrams of the model comb PS set. The boundary for first-harmonic elastic nonlinearity ($\gamma_{0,e}^{*}$) was always located below that for first-harmonic viscous nonlinearity ($\gamma_{0,v}^{*}$), as reported by Song and Hyun [38]. The critical strain amplitudes for first-harmonic nonlinearities of PS290 scaled as $\gamma_{0}^{*}\;\propto$ *ω*^−1^ at low frequency, indicating that the nonlinearities were controlled by strain rate. On increasing frequency, they became constant ($\gamma_{0,e}^{*}$ ≈ 0.50 and $\gamma_{0,v}^{*}$ ≈ 0.52) and then displayed a further increase. Interestingly, the Pipkin diagrams of DC and LB PS exhibited a two-step change of the boundary shape. As an example (Figure 7d), the critical strain amplitudes of PS290-30-44 were constant at a low frequency ($\gamma_{0,e}^{*}$ ≈ 0.81 and $\gamma_{0,v}^{*}$ ≈ 1.23). On increasing the frequency beyond branch relaxation timescale (~1 rad/s), they decreased and reached new constant values ($\gamma_{0,e}^{*}$ ≈ 0.57 and $\gamma_{0,v}^{*}$ ≈ 0.84). Thus, the Pipkin diagrams of DC and LB PS also reflected the hierarchical characteristics in the relaxation processes of these branched polymers. Furthermore, importantly, these results revealed that the linear-to-nonlinear transitions in material response could be controlled by the degree of branching.

**Figure 8:**
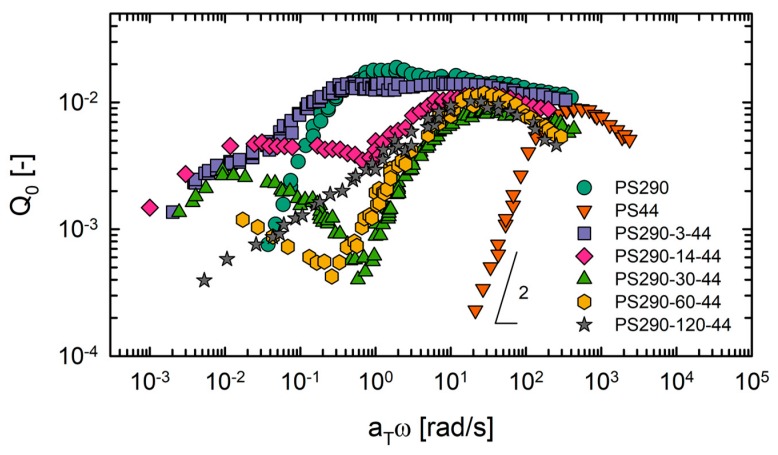
*Q*_0_ master curves of model comb PS set at *T*_ref_ = 180 °C.

Next, we investigated the third-harmonic MAOS nonlinearities of model comb PS set using the *Q*_0_ parameter (Figure 8). The earlier *Q*_0_ studies on model comb PS showed that comb PS with entangled linear branches had two *Q*_0_ peaks—one corresponding to branch relaxation at a higher frequency and the other corresponding to backbone relaxation at a lower frequency [16,30]. Here, the model comb PS used also exhibited two local maxima in *Q*_0_ master curves. Remarkably, the high-frequency *Q*_0_ peak positions of DC PS (~32 rad/s) were not changed by *N*_br_, whereas that of LC PS was located at a lower frequency. The different *Q*_0_ peak of PS290-3-44 originated from the distribution in the number and location of branches, as demonstrated in Section 4.2. In addition, PS290-3-44 corresponded to LC polymer, which exhibited star-like behavior in zero-shear viscosity scaling (Figure 4) [21]. Therefore, the relaxation processes of the backbone and the branches might not be well separated, which can make the high-frequency peak broad (approximately 0.3–20 rad/s). We speculate that this broad high-frequency peak of PS290-3-44 is the combined effect of the backbone and the branches and that the low-frequency peak is due to the relaxation of the remaining effective backbone chain.

Along with the same *Q*_0_ peak position of DC PS near 32 rad/s, the value of this *Q*_0_ peak was almost the same in all DC PS (0.01 ± 0.001). Hyun and Wilhelm [16] showed that, for a comb with the fixed number of branches (~30), longer branches resulted in a higher value of branch *Q*_0_ peak and shifted it toward lower frequency. This indicates that the magnitude and location of high-frequency *Q*_0_ peak of DC polymers are functions of the branch length and are not affected by *N*_br_. The *Q*_0_ maximum of PS44 was also identical to the value corresponding to the branch part but its location was at a higher frequency due to different relaxation processes. PS44 relaxes by reptation combined with CLF, whereas the branches of DC PS relax by only retraction process. Three DC PSs (PS290-14-44, PS290-30-44 and PS290-60-44) exhibited two peaks clearly, which indicates that relaxation processes of the backbone and the branches are well separated in these polymers. The local minimum value was observed near 1 rad/s, where first-harmonic MAOS moduli were divided into backbone and branch contributions.

PS290-120-44 exhibited an unusual *Q*_0_ behavior. This LB PS did not have a *Q*_0_ peak corresponding to the backbone contribution, which contrasted with the fact that first-harmonic MAOS moduli reflected both contributions from the backbone and the branches. We are not aware of the detailed mechanism for this unusual behavior. However, we speculate that the stretched backbone conformation in LB is related to no low-frequency *Q*_0_ peak, which is the critical difference between DC and LB. The stretched backbone conformation might not respond to weakly nonlinear MAOS flow because the backbone is already stretched.

**Figure 9:**
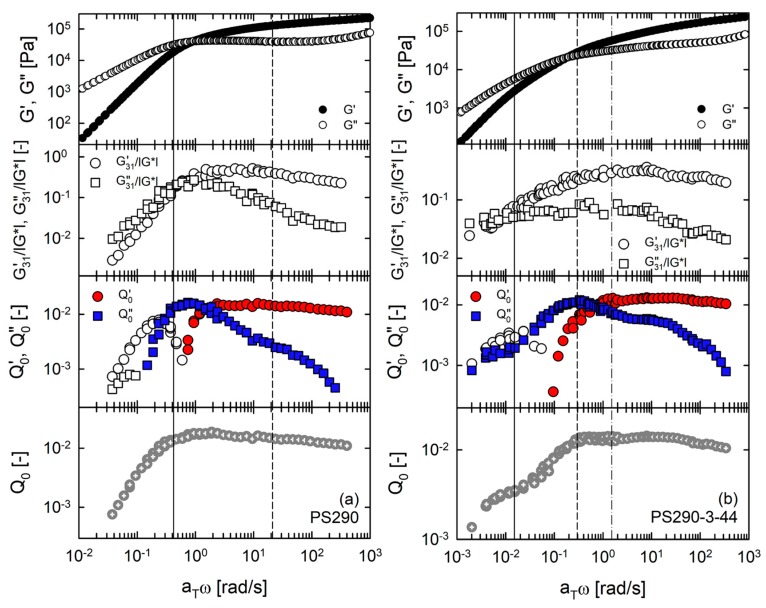

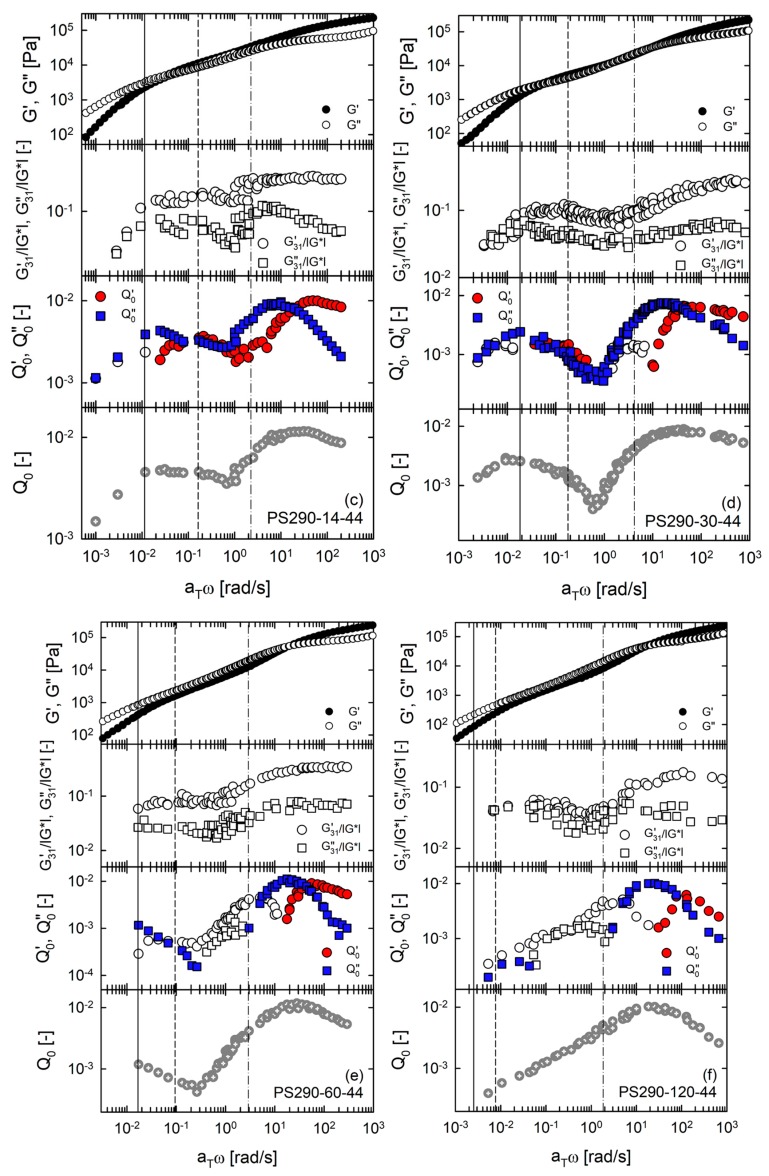
Comparison of linear and nonlinear master curves of model comb PS set with characteristic relaxation times. The solid line indicates the terminal relaxation timescale (*τ*_L_). The dashed line indicates the backbone Rouse timescale (*τ*_R,bb_). The dash-dot line indicates the branch relaxation timescale (*τ*_br_).

Because *Q*_0_ is a combined parameter of two third-harmonic MAOS moduli, we calculated the elastic and viscous parts of *Q*_0_ ($Q_{0}^{\prime }$ and $Q_{0}^{\prime \prime }$). All viscoelastic fluids with a finite relaxation time have a negative sign of third-harmonic nonlinearity in the terminal regime, which is predicted by a fourth-order fluid expansion [46]. Two linear PS exhibited the typical terminal behavior ($Q_{0}^{\prime }\;\propto$ *ω*^3^ and $Q_{0}^{\prime \prime }\;\propto$ *ω*^2^) with a negative sign (see Figure 9 in the next section) [37]. We were not able to experimentally reach the terminal regime of comb and bottlebrush PS because the measurements need to be conducted at a high temperature above 260 °C. However, considering that the absolute values of $Q_{0}^{\prime }$ and $Q_{0}^{\prime \prime }$ decrease with a lower frequency, we expect that the comb and bottlebrush PS display the terminal behavior at lower frequency. In Table 2, we summarized the number of sign changes in $Q_{0}^{\prime }$ and $Q_{0}^{\prime \prime }$ on increasing frequency from the terminal regime to the plateau regime relating to the branches. Based on these sign changes, the MAOS plots can be divided into many regimes. Thus, we counted the number of such regimes. The master curves of $Q_{0}^{\prime }$ and $Q_{0}^{\prime \prime }$ of each sample are presented in Figure 9.

Two linear PS exhibited the same behavior as polymer solutions used in Song and Hyun [37]. $Q_{0}^{\prime }$ and $Q_{0}^{\prime \prime }$ of linear PS showed a sign change once, respectively. Therefore, $Q_{0}^{\prime }$ and $Q_{0}^{\prime \prime }$ plots can be divided into three regimes based on sign changes. $Q_{0}^{\prime }$ and $Q_{0}^{\prime \prime }$ of PS290-3-44 also showed a sign change once, respectively, resulting in 3 regimes in the MAOS plots. The main difference between PS290-3-44 and linear PS was observed in $Q_{0}^{\prime \prime }$. PS290-3-44 had two local peaks of $Q_{0}^{\prime \prime }$, which contributed to the onset of two *Q*_0_ peaks of PS290-3-44. DC and LB PS showed multiple sign changes of $Q_{0}^{\prime }$ and $Q_{0}^{\prime \prime }$. At the frequency corresponding to the rubbery plateau of branches, all DC and LB PS had positive $Q_{0}^{\prime }$ and $Q_{0}^{\prime \prime }$. As the frequency decreased toward the terminal regime, signs of $Q_{0}^{\prime }$ and $Q_{0}^{\prime \prime }$ changed differently depending on *N*_br_. In the frequency range between two rubbery plateau regimes, $Q_{0}^{\prime }$ and $Q_{0}^{\prime \prime }$ of PS290-14-44 did not change signs and are always positive. PS290-14-44 would show sign changes of $Q_{0}^{\prime }$ and $Q_{0}^{\prime \prime }$ to negative value in the terminal regime. Thus, the MAOS master curves of PS290-14-44 are divided into three regimes. PS290-30-44 did not exhibit a sign change of $Q_{0}^{\prime \prime }$ before entering the terminal regime. However, $Q_{0}^{\prime }$ of PS290-30-44 changed its sign three times [(+) → (-) → (+) → (-) with decreasing frequency], which resulted in five regimes in the MAOS plots. In contrast, PS290-60-44 and PS290-120-44 exhibited three sign changes in $Q_{0}^{\prime \prime }$ [(+) → (-) → (+) → (-) with decreasing frequency] while $Q_{0}^{\prime }$ changed sign once. The multiple sign changes in $Q_{0}^{\prime }$ and $Q_{0}^{\prime \prime }$ were not observed when *N*_br_ is small. Currently, we do not have an idea about the origin of these multiple sign changes in third-harmonic MAOS nonlinearities because no molecular mechanism of MAOS nonlinearity has been reported. However, these results reveal that polymer topologies affect signs as well as magnitudes of third-harmonic MAOS nonlinearities, which has never been reported before.

### 4.4. Comparison of Rheological Parameters with Characteristic Relaxation Times

We compared the linear and nonlinear rheological properties with characteristic relaxation times. Three relaxation times were calculated and are listed in Table 3. Terminal relaxation time *τ*_L_ was calculated from linear viscoelastic data as follows: *τ*_L_ = [*G*′/(*ωG*″)]*_ω_*_→0_. Backbone Rouse time *τ*_R,bb_ was calculated based on the hypothesis suggested by Lentzakis, Vlassopoulos, Read, Lee, Chang, Driva and Hadjichristidis [10] that the ratio of the terminal relaxation time to the backbone Rouse time for the comb is the same as the ratio of terminal relaxation time to the Rouse time for an equivalent linear polymer with the same effective entanglement number ($Z_{\mathrm{bb}}^{\mathrm{dil}}$). The effective entanglement number $Z_{\mathrm{bb}}^{\mathrm{dil}}$ of the backbone chain can be calculated by considering the motion of the backbone ends, as discussed in Section 4.2 [14,28,47]. Under the assumption of evenly distributed branch points along the backbone chain, the molecular weight of the end part of backbone is *M*_bb,end_ = *M*_w,bb_/(*N*_br_ + 1), which is the same as the molecular weight of backbone segments between branch points. Then, the effective backbone molecular weight is $M_{w,\mathrm{bb}}^{\mathrm{dil}}$ = *M*_w,bb_ – 2*x*_c_*M*_bb,end_, where *x*_c_ is the fractional length of the backbone-end portion varied by branch size. If branches are longer than backbone ends (*M*_br_ > *M*_bb,end_), *x*_c_ is 1 because backbone ends have fully relaxed on the branch relaxation timescale. Otherwise (*M*_br_ < *M*_bb,end_), *x*_c_ is the ratio of molecular weight of the branch to the molecular weight of the backbone end part (*M*_br_/*M*_bb,end_). Finally, $Z_{\mathrm{bb}}^{\mathrm{dil}}$ of the backbone chain is calculated as follows: $Z_{\mathrm{bb}}^{\mathrm{dil}}$ = ($M_{w,\mathrm{bb}}^{\mathrm{dil}}$/*M*_e_)($M_{w,\mathrm{bb}}^{\mathrm{dil}}$/*M*_w_). Using the calculated $Z_{\mathrm{bb}}^{\mathrm{dil}}$, backbone Rouse time *τ*_R,bb_ was calculated from the formula based on the tube theory [1]: *τ*_R,bb_ = *τ*_L_/(3$Z_{\mathrm{bb}}^{\mathrm{dil}}$). Branch relaxation time *τ*_br_ was obtained from the peak/point of change in slope in the linear viscoelastic phase angle [5,21].

In Figure 9, we plotted linear and nonlinear master curves of model comb PS set, together with the characteristic relaxation times (*τ*_L_, *τ*_R,bb_ and *τ*_br_). PS290 exhibited the maximum of *Q*_0_, $Q_{0}^{\prime \prime }$ and normalized $G_{31}^{\prime \prime }$ ($\equiv G_{31}^{\prime \prime }$/|G*|) near the terminal relaxation time, as reported in earlier studies [28,36,37]. The critical difference between first- and third-harmonic MAOS material functions was observed in the results of PS290-3-44. The normalized $G_{31}^{\prime \prime }$ of PS290-3-44 displayed one plateau-like peak whereas $Q_{0}^{\prime \prime }$ and *Q*_0_ displayed two local peaks reflecting characteristics of hierarchical relaxation. This implies that third-harmonic MAOS reflects the architectural features of polymers more sensitively than first-harmonic MAOS material function. For PS290-3-44, the peaks of *Q*_0_, $Q_{0}^{\prime \prime }$ and $G_{31}^{\prime \prime }$/|G*| were observed near *τ*_R,bb_. The additional peaks of *Q*_0_ and $Q_{0}^{\prime \prime }$ appeared at the frequency corresponding to *τ*_L_, which confirms that the low-frequency peaks are due to the remaining effective backbone chains.

The first-harmonic MAOS moduli of DC and LB exhibited two local peaks when they were normalized by SAOS complex modulus (|G*|). However, two peaks were more evident in the master curves of third-harmonic properties. For DC PS, the high-frequency peaks of *Q*_0_ and $Q_{0}^{\prime \prime }$ relating to branch relaxation appeared at the frequency larger than the inverse of *τ*_br_. In contrast, the low-frequency peaks of *Q*_0_ and $Q_{0}^{\prime \prime }$ relating to backbone relaxation were observed at the frequency corresponding to *τ*_L_. All MAOS parameters of DC PS exhibited the local minimum between *τ*_R,bb_ and *τ*_br_ when their absolute values were used. In addition, featured sign changes of $Q_{0}^{\prime }$ and $Q_{0}^{\prime \prime }$ were observed between these two timescales. In contrast, PS290-120-44 showed the local minimum only in the absolute values of first-harmonic MAOS parameters, while those of third-harmonic MAOS parameters decreased continuously with decreasing frequency. Even at the terminal relaxation timescale, third-harmonic parameters of PS290-120-44 did not display an upturn. Again, we speculate that the stretched backbone conformation in LB is responsible for no low-frequency peak of *Q*_0_. However, it remains an important question why the stretched backbone conformation makes an additional contribution only to the first-harmonic MAOS moduli and not the third-harmonic MAOS moduli. More systematic approaches would be necessary to answer this question.

## 5. Conclusions

We investigated linear and nonlinear oscillatory shear (SAOS and MAOS) rheology of model comb PS melts, where the number of branches along the backbone was controlled while the branch length was kept. The model PS used was classified into three conformational regimes: loosely grafted comb (LC), densely grafted comb (DC) and loosely grafted bottlebrush (LB). In each regime, scaling of zero-shear viscosity *η*_0_ versus total weight-average molecular weight *M*_w_ was different: *η*_0_ ~ exp (*M*_w_) in LC regime (*Z*_s_ > 3), *η*_0_ ~ $M_{w}^{-3.4}$ in DC regime with an entangled, diluted backbone (1 < *Z*_s_ < 3), *η*_0_ ~ $M_{w}^{-1}$ in DC regime with an unentangled, diluted backbone (0.2 < *Z*_s_ < 1) and *η*_0_ ~ $M_{w}^{5}$ in LB regime (*Z*_s_ < 0.2). The plateau modulus of the diluted backbone scaled as *G*_N,bb_ ~ $M_{w}^{-2}$, irrespective of the comb and bottlebrush conformations [21].

Linear viscoelastic properties of model comb PS set were compared with predictions of the hierarchical model. For PS290-3-44, deviations between the experimental data and the model originated from the statistical distribution of the number and location of branches. Thus, we discussed the effect and importance of this distribution on relaxation processes. For quantitative comparison, η_0_ and *G*_N,bb_ of the experimental data and the model predictions were plotted as functions of *M*_w_. The model failed to predict *η*_0_ and *G*_N,bb_ of PS290-60-44 and PS290-120-44 because the model excludes the effect of side chain crowding in the densely branched topologies.

We presented the first-harmonic MAOS moduli of comb and bottlebrush polymers for the first time. The first-harmonic MAOS data reflected the hierarchy of relaxation processes in comb topologies well. DC and LB PS exhibited two plateaus in $G_{31}^{\prime }$ and $G_{31}^{\prime \prime }$; one contributed from branches at a higher frequency and the other contributed from backbone at a lower frequency. When the plateau values were plotted as functions of *M*_w_, the high-frequency plateaus were constant whereas the low-frequency plateaus scaled with $M_{w}^{-2}$, indicating that first-harmonic MAOS moduli were also affected by dynamic tube dilution (DTD) effect. Accordingly, the boundary for linear-to-nonlinear transitions (Pipkin diagram) of DC and LB PS exhibited a two-step change. Relative intrinsic nonlinearity *Q*_0_ and its viscous part $Q_{0}^{\prime \prime }$ (third-harmonic MAOS material functions) also showed two peaks for LC and DC PS reflecting the hierarchical relaxation processes. In contrast, the low-frequency *Q*_0_ peak corresponding to the backbone part of LB disappeared. The stretched backbone conformation in LB might be related to this no low-frequency *Q*_0_ peak. Comparison of MAOS data with characteristic relaxation times (terminal relaxation time, backbone Rouse time and branch relaxation time) supported the discussion on changes in the number of peaks of relative nonlinearities.

The findings from the first- and third-harmonic MAOS rheology of LC PS indicate that third-harmonic MAOS reflects structural features of different topologies better than first-harmonic MAOS. PS290-3-44 (LC) has a hierarchical relaxation process due to the averagely three branches. However, first-harmonic MAOS moduli displayed one broad peak while third-harmonic MAOS properties exhibited two peaks clearly. Nevertheless, it is necessary to characterize both first- and third-harmonic MAOS properties of polymers with complex architectures because PS290-120-44 (LB) reflected the feature of hierarchical relaxation in first-harmonic MAOS but the stretched conformation of backbone in third-harmonic MAOS.

## Figures and Tables

**Table 1:** Molecular characteristics of model comb polystyrene (PS) set.

Name ^a^	*M*_w,bb_^b^ (kg/mol)	PDI_bb_^b^ (-)	*M*_w,br_^b^ (kg/mol)	PDI_br_^b^ (-)	*M*_w_^b^ (kg/mol)	PDI ^b^ (-)	*Z*_s_^c^ (-)	*N*_br_^c^ (-)	Topology ^d^
PS290	290	1.07			290	1.07	20	0	Linear
PS44	43	1.03			43	1.03	3.03	0	Linear
PS290-3-44	290	1.10	45	1.07	420	1.11	5.06	3	LC
PS290-14-44	290	1.10	45	1.07	900	1.08	1.36	14	DC
PS290-30-44	290	1.10	43	1.03	1600	1.03	0.65	30	DC
PS290-60-44	290	1.10	44	1.05	2900	1.03	0.33	60	DC
PS290-120-44	290	1.10	44	1.05	5570	1.11	0.17	120	LB

^a^ Sample nomenclature is as follows: PS*M*_w,bb_-*N*_br_-*M*_w,br._^b^ Weigh-average molecular weight and polydispersity index of backbone, branch and combs measured by SEC-MALLS. ^c^ Calculated by *N*_br_ = (*M*_w_ – *M*_w,bb_)/*M*_w,br_ and *Z*_s_ = *Z*_bb_/(*N*_br_ + 1) where *Z*_bb_ = (*M*_w,bb_/*M*_e_) ≅ 20 and *M*_e_ = 14.4 kg/mol. ^d^ Topologies of comb and bottlebrush polymers were determined using scaling analysis. LC: loosely grafted comb; DC: densely grafted comb; LB: loosely grafted bottlebrush.

**Table 2:** The number of sign changes in $Q_{0}^{\prime }$ and $Q_{0}^{\prime \prime }$ on increasing frequency from the terminal regime to the plateau regime relating to the branches and the total number of resultant regimes.

Samples	$Q_{0}^{\prime }$	$Q_{0}^{\prime }$	# of Regimes
PS290	1	1	3
PS44	1	1	3
PS290-3-44	1	1	3
PS290-14-44	1	1	3
PS290-30-44	3	1	5
PS290-60-44	1	3	5
PS290-120-44	1	3	5

**Table 3:** Characteristic relaxation times of model comb PS set at *T*_ref_ = 180 °C.

Samples	*τ*_L_^a^ (s)	$Z_{bb}^{dil}$ (-)	*τ*_R,bb_^d^ (s)	*τ*_br_^e^ (s)
PS290	2.37 × 10^0^	20.14 ^b^	3.91 × 10^−2^	-
PS44	2.34 × 10^−3^	3.06 ^b^	2.55 × 10^−4^	-
PS290-3-44	6.63 × 10^1^	6.54 ^c^	3.38 × 10^0^	6.58 × 10^−1^
PS290-14-44	8.81 × 10^1^	4.80 ^c^	6.12 × 10^0^	4.42 × 10^−1^
PS290-30-44	5.46 × 10^1^	3.25 ^c^	5.60 × 10^0^	2.40 × 10^−1^
PS290-60-44	5.96 × 10^1^	1.88 ^c^	1.06 × 10^1^	3.47 × 10^−1^
PS290-120-44	3.96 × 10^2^	1.01 ^c^	1.30 × 10^2^	5.44 × 10^−1^

^a^ Terminal relaxation time *τ*_L_ = [*G*′/(*ωG*″)]*_ω_*_→0_. ^b^ For linear PS, $Z_{\mathrm{bb}}^{\mathrm{dil}}$ = (*M*_w_/*M*_e_), where *M*_e_ = 14.4 kg/mol. ^c^ For comb and bottlebrush PS, $Z_{\mathrm{bb}}^{\mathrm{dil}}$ = ($M_{w,\mathrm{bb}}^{\mathrm{dil}}$/*M*_e_)($M_{w,\mathrm{bb}}^{\mathrm{dil}}$/*M*_w_), where $M_{w,\mathrm{bb}}^{\mathrm{dil}}$ = *M*_w,bb_ – 2*x*_c_*M*_bb,end_. *M*_bb,end_ = *M*_w,bb_/(*N*_br_ + 1) and *x*_c_ is 1 (*M*_br_ > *M*_bb,end_) or *M*_br_/*M*_bb,end_ (*M*_br_ < *M*_bb,end_). ^d^ Backbone Rouse time *τ*_R,bb_ = *τ*_L_/(3$Z_{\mathrm{bb}}^{\mathrm{dil}}$). ^e^ Branch relaxation time *τ*_br_ was obtained from the peak/point of change in slope in the linear viscoelastic phase angle.

## References

[1] Dealy J.M., Larson R.G. (2006). Structure and Rheology of Molten Polymers: From Structure to Flow Behavior and Back Again.

[2] Wood-Adams P.M., Dealy J.M., deGroot A.W., Redwine O.D. (2000). Effect of molecular structure on the linear viscoelastic behavior of polyethylene. Macromolecules.

[3] Hatzikiriakos S.G. (2000). Long chain branching and polydispersity effects on the rheological properties of polyethylenes. Polym. Eng. Sci..

[4] Abbasi M., Ebrahimi N.G., Nadali M., Esfahani M.K. (2012). Elongational viscosity of LDPE with various structures: Employing a new evolution equation in MSF theory. Rheol. Acta.

[5] Lentzakis H., Das C., Vlassopoulos D., Read D.J. (2014). Pom-pom-like constitutive equations for comb polymers. J. Rheol..

[6] Snijkers F., Pasquino R., Olmsted P., Vlassopoulos D. (2015). Perspectives on the viscoelasticity and flow behavior of entangled linear and branched polymers. J. Phys. Condens. Mat..

[7] Larson R.G. (2001). Combinatorial rheology of branched polymer melts. Macromolecules.

[8] Fetters L.J., Kiss A.D., Pearson D.S., Quack G.F., Vitus F.J. (1993). Rheological behavior of star-shaped polymers. Macromolecules.

[9] van Ruymbeke E., Kapnistos M., Vlassopoulos D., Huang T., Knauss D.M. (2007). Linear melt rheology of pom-pom polystyrenes with unentangled branches. Macromolecules.

[10] Lentzakis H., Vlassopoulos D., Read D.J., Lee H., Chang T., Driva P., Hadjichristidis N. (2013). Uniaxial extensional rheology of well-characterized comb polymers. J. Rheol..

[11] Lee J.H., Orfanou K., Driva P., Iatrou H., Hadjichristidis N., Lohse D.J. (2008). Linear and nonlinear rheology of dendritic star polymers: Experiment. Macromolecules.

[12] Roovers J. (1979). Synthesis and dilute solution characterization of comb polystyrenes. Polymer.

[13] Stadler F.J., Arikan-Conley B., Kaschta J., Kaminsky W., Münstedt H. (2011). Synthesis and characterization of novel ethylene-*graft*-ethylene/propylene copolymers. Macromolecules.

[14] Kapnistos M., Vlassopoulos D., Roovers J., Leal L.G. (2005). Linear rheology of architecturally complex macromolecules: Comb polymers with linear backbones. Macromolecules.

[15] Snijkers F., Vlassopoulos D., Ianniruberto G., Marrucci G., Lee H., Yang J., Chang T. (2013). Double stress overshoot in start-up of simple shear flow of entangled comb polymers. ACS Macro Lett..

[16] Hyun K., Wilhelm M. (2009). Establishing a new mechanical nonlinear coefficient *Q* from FT-rheology: First investigation of entangled linear and comb polymer model systems. Macromolecules.

[17] Paturej J., Sheiko S.S., Panyukov S., Rubinstein M. (2016). Molecular structure of bottlebrush polymers in melts. Sci. Adv..

[18] Daniel W.F.M., Burdyńska J., Vatankhah-Varnoosfaderani M., Matyjaszewski K., Paturej J., Rubinstein M., Dobrynin A.V., Sheiko S.S. (2016). Solvent-free, supersoft and superelastic bottlebrush melts and networks. Nat. Mater..

[19] Liang H., Morgan B.J., Xie G., Martinez M.R., Zhulina E.B., Matyjaszewski K., Sheiko S.S., Dobrynin A.V. (2018). Universality of the entanglement plateau modulus of comb and bottlebrush polymer melts. Macromolecules.

[20] Dalsin S.J., Hillmyer M.A., Bates F.S. (2015). Linear rheology of polyolefin-based bottlebrush polymers. Macromolecules.

[21] Abbasi M., Faust L., Riazi K., Wilhelm M. (2017). Linear and extensional rheology of model branched polystyrenes: From loosely grafted combs to bottlebrushes. Macromolecules.

[22] Abbasi M., Faust L., Wilhelm M. (2019). Comb and bottlebrush polymers with superior rheological and mechanical properties. Adv. Mater..

[23] Hu M., Xia Y., McKenna G.B., Kornfield J.A., Grubbs R.H. (2011). Linear rheological response of a series of densely branched brush polymers. Macromolecules.

[24] Haugan I.N., Maher M.J., Chang A.B., Lin T.-P., Grubbs R.H., Hillmyer M.A., Bates F.S. (2018). Consequences of grafting density on the linear viscoelastic behavior of graft polymers. ACS Macro Lett..

[25] López-Barrón C.R., Shivokhin M.E. (2019). Extensional strain hardening in highly entangled molecular bottlebrushes. Phys. Rev. Lett..

[26] López-Barrón C.R., Shivokhin M.E., Hagadorn J.R. (2019). Extensional rheology of highly-entangled α-olefin molecular bottlebrushes. J. Rheol..

[27] Cziep M.A., Abbasi M., Heck M., Arens L., Wilhelm M. (2016). Effect of molecular weight, polydispersity and monomer of linear homopolymer melts on the intrinsic mechanical nonlinearity ^3^*Q*_0_(*ω*) in MAOS. Macromolecules.

[28] Song H.Y., Park S.J., Hyun K. (2017). Characterization of dilution effect of semidilute polymer solution on intrinsic nonlinearity *Q*_0_ via FT rheology. Macromolecules.

[29] Song H.Y., Nnyigide O.S., Salehiyan R., Hyun K. (2016). Investigation of nonlinear rheological behavior of linear and 3-arm star 1,4-cis-polyisoprene (PI) under medium amplitude oscillatory shear (MAOS) flow via ft-rheology. Polymer.

[30] Kempf M., Ahirwal D., Cziep M., Wilhelm M. (2013). Synthesis and linear and nonlinear melt rheology of well-defined comb architectures of PS and PpMS with a low and controlled degree of long-chain branching. Macromolecules.

[31] Wilhelm M. (2002). Fourier-transform rheology. Macromol. Mater. Eng..

[32] Giacomin A.J., Dealy J.M., Collyer A.A. (1993). Large-amplitude oscillatory shear. Techniques in Rheological Measurement.

[33] Hyun K., Wilhelm M., Klein C.O., Cho K.S., Nam J.G., Ahn K.H., Lee S.J., Ewoldt R.H., McKinley G.H. (2011). A review of nonlinear oscillatory shear tests: Analysis and application of large amplitude oscillatory shear (LAOS). Prog. Polym. Sci..

[34] Pearson D.S., Rochefort W.E. (1982). Behavior of concentrated polystyrene solutions in large-amplitude oscillating shear fields. J. Polym. Sci. Polym. Phys. Ed..

[35] Ewoldt R.H., Bharadwaj N.A. (2013). Low-dimensional intrinsic material functions for nonlinear viscoelasticity. Rheol. Acta.

[36] Song H.Y., Hyun K. (2019). First-harmonic intrinsic nonlinearity of model polymer solutions in medium amplitude oscillatory shear (MAOS). Korea-Aust. Rheol. J..

[37] Song H.Y., Hyun K. (2018). Decomposition of *Q*_0_ from FT-rheology into elastic and viscous parts: Intrinsic-nonlinear master curves for polymer solutions. J. Rheol..

[38] Song H.Y., Hyun K. (2019). Nonlinear material functions under medium amplitude oscillatory shear (MAOS) flow. Korea-Aust. Rheol. J..

[39] Song H.Y., Salehiyan R., Li X., Lee S.H., Hyun K. (2017). A comparative study of the effects of cone-plate and parallel-plate geometries on rheological properties under oscillatory shear flow. Korea-Aust. Rheol. J..

[40] Park S.J., Shanbhag S., Larson R.G. (2005). A hierarchical algorithm for predicting the linear viscoelastic properties of polymer melts with long-chain branching. Rheol. Acta.

[41] Wang Z., Chen X., Larson R.G. (2010). Comparing tube models for predicting the linear rheology of branched polymer melts. J. Rheol..

[42] Das C., Inkson N.J., Read D.J., Kelmanson M.A., McLeish T.C.B. (2006). Computational linear rheology of general branch-on-branch polymers. J. Rheol..

[43] Ahmadi M., Bailly C., Keunings R., Nekoomanesh M., Arabi H., van Ruymbeke E. (2011). Time marching algorithm for predicting the linear rheology of monodisperse comb polymer melts. Macromolecules.

[44] Chen X., Larson R.G. (2008). Effect of branch point position on the linear rheology of asymmetric star polymers. Macromolecules.

[45] Hyun K., Kim S.H., Ahn K.H., Lee S.J. (2002). Large amplitude oscillatory shear as a way to classify the complex fluids. J. Non-Newtonian Fluid Mech..

[46] Bharadwaj N.A., Ewoldt R.H. (2014). The general low-frequency prediction for asymptotically nonlinear material functions in oscillatory shear. J. Rheol..

[47] Kirkwood K.M., Leal L.G., Vlassopoulos D., Driva P., Hadjichristidis N. (2009). Stress relaxation of comb polymers with short branches. Macromolecules.

